# Methodological Challenges in Protein Microarray and Immunohistochemistry for the Discovery of Novel Autoantibodies in Paediatric Acute Disseminated Encephalomyelitis

**DOI:** 10.3390/ijms18030679

**Published:** 2017-03-22

**Authors:** Patrick Peschl, Melanie Ramberger, Romana Höftberger, Karin Jöhrer, Matthias Baumann, Kevin Rostásy, Markus Reindl

**Affiliations:** 1 Clinical Department of Neurology, Medical University of Innsbruck, Innsbruck A-6020, Austria; patrick.peschl@i-med.ac.at (P.P.); ramberger.melanie@gmail.com (M.R.); 2 Institute of Neurology, Medical University of Vienna, Vienna A-1090, Austria; romana.hoeftberger@meduniwien.ac.at; 3 Tyrolean Cancer Research Institute, Innsbruck A-6020, Austria; karin.joehrer@tkfi.at; 4 Division of Paediatric Neurology, Department of Paediatrics I, Medical University of Innsbruck, Innsbruck A-6020, Austria; matthias.baumann@tirol-kliniken.at; 5 Department of Paediatric Neurology, Witten/Herdecke University, Children’s Hospital Datteln, Datteln D-45711, Germany; k.rostasy@kinderklinik-datteln.de

**Keywords:** acute disseminated encephalomyelitis, paediatric, autoantibody, autoantigen, protein microarray, immunohistochemistry

## Abstract

Acute disseminated encephalomyelitis (ADEM) is a rare autoimmune-mediated demyelinating disease affecting mainly children and young adults. Differentiation to multiple sclerosis is not always possible, due to overlapping clinical symptoms and recurrent and multiphasic forms. Until now, immunoglobulins reactive to myelin oligodendrocyte glycoprotein (MOG antibodies) have been found in a subset of patients with ADEM. However, there are still patients lacking autoantibodies, necessitating the identification of new autoantibodies as biomarkers in those patients. Therefore, we aimed to identify novel autoantibody targets in ADEM patients. Sixteen ADEM patients (11 seronegative, 5 seropositive for MOG antibodies) were analysed for potential new biomarkers, using a protein microarray and immunohistochemistry on rat brain tissue to identify antibodies against intracellular and surface neuronal and glial antigens. Nine candidate antigens were identified in the protein microarray analysis in at least two patients per group. Immunohistochemistry on rat brain tissue did not reveal new target antigens. Although no new autoantibody targets could be found here, future studies should aim to identify new biomarkers for therapeutic and prognostic purposes. The microarray analysis and immunohistochemistry methods used here have several limitations, which should be considered in future searches for biomarkers.

## 1. Introduction

Acute disseminated encephalomyelitis (ADEM) is an autoimmune disorder of the central nervous system (CNS) mainly affecting children and young adults. According to the International Paediatric Multiple Sclerosis Study Group (IPMSSG), diagnosis of the disease requires a polysymptomatic clinical presentation including focal neurological and encephalopathic symptoms, such as confusion, excessive irritability, lethargy, or coma [1]. The disease course is mainly monophasic with partial or complete recovery, but also multiphasic forms of ADEM have been observed with a recurrence of the initial symptoms or more distinct ADEM episodes appearing at least three months after the first event, making the differentiation to multiple sclerosis (MS) not always simple [2]. Therefore, the identification of disease specific biomarkers is a very important research aim.

One potential biomarker is an autoantibody against myelin oligodendrocyte glycoprotein (MOG), which is located on the outermost surface of myelin sheaths and oligodendrocytes. Several studies found a higher frequency of serum anti-MOG IgG antibodies (MOG antibodies) in paediatric patients with ADEM and other acquired demyelinating diseases, and a significant titre decrease within a period of 12 months in ADEM cases [3,4,5,6,7,8,9,10,11]. Baumann and colleagues found that 58% of investigated ADEM patients were positive for MOG antibodies at disease onset, with a significantly better recovery compared to seronegative patients [12]. However, a considerable group of ADEM patients are seronegative for MOG antibodies, usually also responding to anti-inflammatory treatment regimens or plasmapheresis, indicating the role of other yet unknown autoantibodies in the pathogenesis of the disease.

The aim of this study was therefore to search for novel autoantibodies in MOG antibody seronegative ADEM. For this purpose we analysed sera from ADEM patients by a protein microarray technique, which allows us to investigate 9375 potential antigens. In addition, we used immunohistochemistry (IHC) based on frozen rat brain sections to include a broad range of neuronal and glial surface and intracellular antigens.

## 2. Results

### 2.1. Protein Microarray Did Not Discriminate between Paediatric ADEM Patients with and without MOG Antibodies

**Figure 1:**
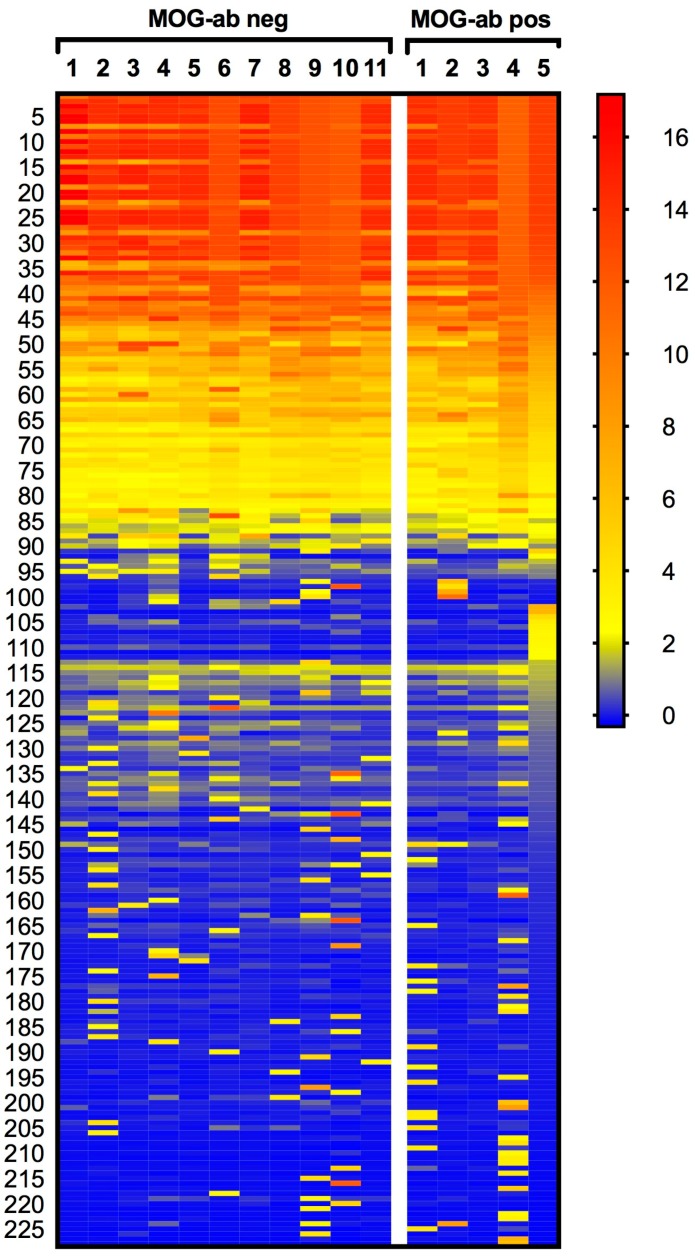
Heatmap of 228 proteins (see Table S1) with significant hits (z-score ≥ 2.33) in at least one serum sample. Columns are individual serum samples from myelin oligodendrocyte glycoprotein antibody (MOG-ab) negative (neg, *n =* 11) and positive (pos, *n =* 5) acute disseminated encephalomyelitis (ADEM) patients; rows are proteins with a z-score ≥ 2.33. Values range from blue (−0.32) to yellow (2.33) to red (17), with a legend on the right side.

**Figure 2:**
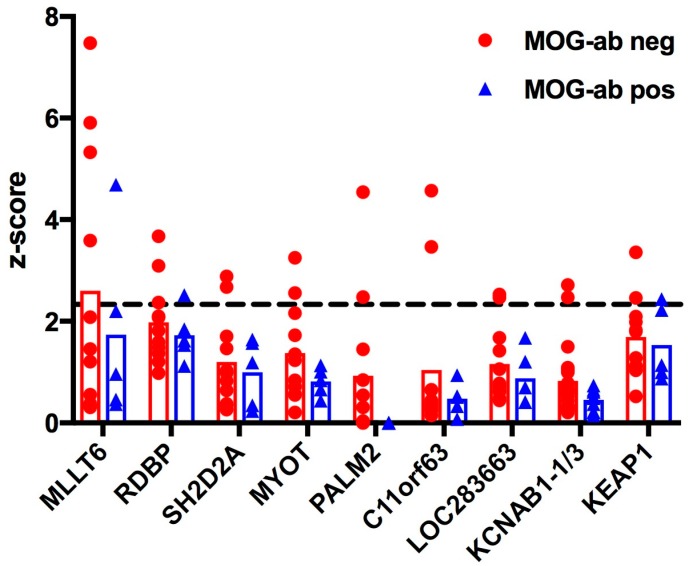
MOG antibody seropositivity does not significantly influence z-scores in paediatric patients with ADEM. Z-scores of candidate proteins (Y-axis) that exceeded the cut-off (2.33) in at least two patients per group are shown (for more details see Table 1). Means are indicated by the bars. The cut-off is shown as a dashed horizontal line. No significant differences were observed (matched two-way ANOVA). MLLT6: Myeloid/lymphoid or mixed-lineage leukaemia, translocated to, 6; RDBP: Negative elongation factor E (NELF-E); MYOT: Myotilin; PALM2: Paralemmin-2; C11orf63: Chromosome 11 open reading frame 63; LOC283663: hypothetical protein LOC283663; KCNAB1-1/3: Voltage-gated potassium channel subunit beta-1, variants 1 and 3; KEAP1: Kelch-like ECH-associated protein 1.

As a first step, we investigated autoantibody profiles in paediatric ADEM patients by applying sera from five patients with serum MOG antibodies and 11 without serum MOG antibodies to a protein ProtoArray^®^ (Invitrogen, Carlsbad, CA, USA) containing 9375 potential human autoantigens. Figure S1 shows an example of a protein ProtoArray^®^ scan. The cut-off z-score was defined as ≥2.33, corresponding to the top 1% quantile of all signals. A total of 228 proteins were identified as targets across all serum samples in at least one patient (Figure 1 and Table S1). Among these, 79 proteins were increased in all 16 samples. However, these proteins included mainly sequences of the immunoglobulin (Ig) family, which can be attributed to unspecific binding. All details to antigen-antibody reactivity are shown in Table S1. In the MOG antibody negative group, nine specific candidate proteins emerged having z-scores above the cut-off (≥2.33) in at least two patients per group (Table 1). The potassium channel KCNAB1 was included as a candidate antigen since two samples showed reactivity above the cut-off to variant 1 (v1) and v3 of KCNAB1, respectively. Although all mean z-scores of those nine proteins were higher in the MOG antibody seronegative patient group, none of them were significantly different between the two patient groups (Figure 2). Besides KCNAB1, we found an additional reactivity of ≥2.33 to voltage-gated potassium channel subunit beta-2, transcript variant 1 (KCNAB2 v1), potassium channel tetramerisation domain containing 18 (KCTD18), and potassium channel tetramerisation domain containing 6 (KCTD6). Each one of these proteins was increased in at least one patient of the MOG antibody negative patient group.

### 2.2. Rat Brain Immunohistochemistry Revealed No Potential Novel Target in Paediatric ADEM

To enlarge the range of potential neuronal and glial surface and intracellular antigens, we also applied the sera to rat brain sections. All tested sera were negative for antibodies to neuronal surface and cerebellar intracellular antigens. Two out of five MOG antibody positive patients showed a positive myelin staining in rat brain sections, thereby confirming the validity of the method. The lack of reactivity in the remaining three MOG antibody positive patients can presumably be attributed to species-dependent differential binding patterns [13]. A representative staining of a MOG antibody positive sample is shown in Figure S2. 

### 2.3. Sera of Paediatric MOG Antibody Negative ADEM Patients Did Not Contain Antibodies against Leucine-Rich Glioma-Inactivated 1 (LGI1) and Contactin-Associated Protein-2 (CASPR2)

Since we identified the potassium channel related proteins KCNAB1, KCNAB2, KCTD6, and KCTD18 as potential target antigens in the group of MOG antibody negative ADEM patients, we analysed all samples for antibodies against voltage gated potassium channel (VGKC) associated proteins leucine-rich glioma-inactivated 1 (LGI1) and contactin-associated protein-2 (CASPR2), which have been reported in subgroups of paediatric inflammatory neurological diseases [14,15]. However, in our cohort of paediatric ADEM patients we did not find any reactivity against the extracellular domains of these VGKC associated proteins.

### 2.4. Antibodies against Recombinant Myelin Proteins/Peptides Did Not Reveal Significant Differences between the Two Groups

Reactivity to myelin peptides was analysed using enzyme-linked immunosorbent assay (ELISA). Antibody responses against rhMOG(1–125), myelin basic protein (MBP), and synthetic myelin peptides (MOG(1–20), MOG(35–55), MBP(13–32), MBP(83–99), MBP(111–129), MBP(146–170), and proteolipid protein PLP(139–154)) were not significantly altered between patients with MOG antibodies and the seronegative group (Figure S3). As controls, we included myelin peptide antibody reactivity from 10 adult MS patients which was recently published [16].

## 3. Discussion

Autoantibodies in demyelinating and other inflammatory neurological diseases are of scientific and clinical interest, and are often associated with pathogenetic events, such as aquaporin-4 (AQP4) autoantibodies in neuromyelitis optica spectrum disorders (NMOSD) [17]. The identification of MOG antibodies as potential biological markers for ADEM motivated several research groups to search for new antibodies against myelin and non-myelin antigens in the CNS [18,19]. For this reason, we aimed to analyse paediatric MOG-negative ADEM patients for potential new autoantibodies with a high throughput method and IHC. We indeed found antibody reactivity to several candidate proteins in MOG antibody seronegative ADEM patients, but none of them was significantly different to the seropositive group. However, due to the small patient numbers, results from statistical analysis should be considered preliminary. Moreover, there may be various rare autoantibodies present only in very few patients and much larger studies would be needed to identify these.

A further limitation of our study was the lack of MOG as antigen on the protein array, although it was claimed to be included on the protein array by a previous publication of Querol et al. using the same protein array [20]. Therefore, MOG antibodies from our seropositive cohort could not be used as a positive control. Likewise, many other neuronal and glial antigens—such as CASPR2 or AQP4—are not present in the protein microarray. Another limitation could be a lack of sensitivity of the assay, due to high background values. However, this was excluded by compensatory statistics. Nevertheless, sensitivity of this microarray was confirmed by the detection of cyclic citrullinated peptide (CCP) antibodies, an established biomarker for rheumatoid arthritis in one patient. Since this patient did not (yet) show any clinical symptoms related to rheumatoid arthritis, and since CCP antibody levels of this patient were below the diagnostic cut-off in a validated ELISA (data not shown), it is likely that the cut-off z-score (2.33) used here might be below the clinically relevant threshold.

The spotting of native proteins in a microarray format does not preserve all original conformational epitopes, which could yield false-negative results, and therefore missing antibodies to conformational epitopes. The importance of an intact conformation to detect disease-related antibodies has been evidenced previously, when MOG antibodies were falsely identified as a prognostic biomarker for MS, but were observed also in several healthy control samples, using recombinant or denatured protein [21,22]. In our study, we also detected antibody responses to MOG, MBP, and PLP peptides in both MOG antibody negative and positive ADEM groups with no significant differences. A previous microarray study [18] analysing antibody reactivity to myelin peptides in patients with ADEM and paediatric/adult MS demonstrated distinct profiles of myelin antibody reactivity in these two groups, specifically autoantibodies of the immunoglobulin G (IgG) isotype to MBP and myelin-associated oligodendrocyte basic protein (MOBP) in ADEM patients. However, in our study we could not confirm these results when comparing ADEM and adult MS patients. 

The finding of two MOG antibody negative patients with reactivity to KCNAB1 and KCNAB2, members of the VGKC-superfamily, and also members of the potassium channel tetramerisation domain (KCTD18, KCTD6), prompted us to test for antibodies against VGKC associated proteins LGI1 and CASPR2, due to a reported association with inflammatory neurologic diseases in children [14,15]. However, testing against VGKC associated proteins LGI1 and CASPR2 did not reveal any positive results. These findings are in line with a previous study [23] in which only a subset of VGKC antibodies also bound LGI1 and CASPR2, whereas the other antibodies targeted intracellular epitopes and non-neuronal targets. 

Additional proteins listed in Table 1 are of intracellular origin (Source: Uniprot), of unknown function or not directly associated with the CNS, and therefore not of interest as relevant targets in this study. Furthermore, it is very likely that these antibodies to intracellular proteins are part of the normal immune repertoire (natural autoantibodies) and only a side effect of cellular degradation processes.

Although we aimed to increase the number of possible intracellular and surface neuronal and glial antigens by using IHC for antibody screening, we could not detect possible antibodies to conformational epitopes with this method, which is commonly used to detect antibodies to intracellular and surface neuronal targets. One potential cause could be the species-specific alteration of amino acid sequences within the recognized antigen epitopes [13]. This is also underlined by the fact that only 2/5 MOG antibody positive patients showed a specific myelin staining on rat tissue.

The use of cerebrospinal fluid (CSF) for screening could have yielded further novel candidate autoantigens, and CSF antibody testing is highly recommended for antibody-mediated neurological autoimmune diseases such as autoimmune encephalitis [24]. On the other hand, MOG antibodies are only rarely found in CSF of ADEM patients, suggesting that there is no intrathecal synthesis of the antibodies [25]. It therefore remains speculative if CSF is superior for antibody screening in the patients investigated here.

## 4. Materials and Methods 

### 4.1. Patients and Samples

Patients’ serum samples were collected in the Clinical Department of Neurology Innsbruck between 2009 and 2013 and stored at −80 °C until use. All patients were diagnosed with a definite monophasic ADEM. The diagnosis was based on the diagnostic criteria of the IPMSSG with clinical assessments according to clinical CNS events, with a probable inflammatory demyelinating cause, and encephalopathy not related to fever, systemic illness, or postictal symptoms [1]. Abnormal cerebral and spinal cord lesions, not indicative of other CNS diseases, were diagnosed by magnetic resonance imaging (MRI). Laboratory tests included cell counts and Ig levels in the CSF. All serum samples analysed in this study were drawn at the acute demyelinating event before immunosuppressive therapy was initiated (baseline). In five of 16 patients, antibodies against MOG were found at baseline [12], with decreasing titres after the first follow up (median age: 4.7 years (range: 2.98–7.01), f:m = 2:3). Seronegative patients (median age 6.2 years (range: 1.13–13.51), f:m = 4:7) had no symptomatic differences compared to MOG antibody seropositive patients. This study was approved by the Ethical Committee of the Medical University of Innsbruck (study number AM4059). All parents and patients (older than 12 years) gave written informed consent to the study protocol.

### 4.2. Protein Microarray

Before use, all serum samples were thawed at room temperature (RT) and centrifuged at 3000× *g* for 10 min at 4 °C.

In total, 16 serum samples (11 MOG antibody negative, 5 MOG antibody positive) were used for the ProtoArray^®^ Human Protein Microarray v5.0 (Invitrogen, Carlsbad, CA, USA). These microarrays contain duplicates of 9375 human full-length native proteins, expressed as N-terminal glutathione *S*-transferase (GST) fusion proteins using a baculovirus-based expression system. ProtoArrays^®^ were handled according to the manufacturer’s guidelines and recommendations. Briefly, slides were equilibrated at 4 °C and left on ice for the whole procedure. All buffers, reagents, and samples were cooled down to 4 °C before usage and handled under sterile conditions. Slides were incubated with Blocking Buffer (50 mM HEPES (Sigma-Aldrich, Saint Louis, MO, USA), pH 7.5, 200 mM NaCl (Merck, Darmstadt, Germany), 0.08% Triton^®^ X-100 (Merck), 25% Glycerol (Sigma-Aldrich), 20 mM reduced glutathione (Sigma-Aldrich), 1× Synthetic Block (Sigma-Aldrich), 1 mM Dithiothreitol (Sigma-Aldrich)) for 1 h with gentle agitation. After aspiration of Blocking Buffer, 5 mL of diluted serum (1:500 in Washing Buffer (1× phosphate buffer saline (PBS), 0.1% Tween 20, 1× Synthetic Block (all Sigma-Aldrich)) were added to each slide and incubated for 90 min with gentle agitation. Arrays were then washed five times with Washing Buffer for 5 min. Human bound antibodies were labelled with Alexa Fluor^®^ 647 goat anti human IgG antibody (Life Technologies, Carlsbad, CA, USA) 1:2000 in Washing Buffer for 90 min with gentle shaking, followed by five washing steps. Finally, arrays were rinsed with deionized water and centrifuged at 200× *g* for 1 min. ProtoArrays^®^ were scanned with a GenePix 4000B array scanner (Axon Instruments Inc., Union City, CA, USA) at a wavelength of 635 nm. Images were analysed and the array grid was acquired with the Prospector Imager v5.2.3 software (Invitrogen).

### 4.3. Rat Brain Immunohistochemistry

All serum samples were analysed for IgG antibodies directed either to surface (neuropil staining) or intracellular antigens by IHC on snapfrozen rat brain tissue as described previously, methods that are optimized to detect surface or intracellular unknown antibody targets [26]. In brief, rats were sacrificed with CO_2_ and underwent two different protocols for antigen staining. For the surface staining, native brain was dissected, sagittally sectioned, and fixed with 4% PFA (paraformaldehyde) (Affymetrix, Santa Clara, CA, USA) in 1× PBS (Morphisto, Frankfurt am Main., Germany) for 1 h at 4 °C. Brain hemispheres were then cryoprotected with 40% sucrose (Merck) for 48 h at 4 °C, subsequently embedded in freezing medium (O.C.T.™, Tissue-Tek^®^, Sakura, Alphen aan den Rijn, The Netherlands), and quickly frozen with liquid nitrogen pre-chilled methylbutan (Sigma-Aldrich). 

Frozen sections (7 μm) were stored at −20 °C until use, and then thawed at RT for 20 min, washed once with PBS, and incubated with 0.3% hydrogen peroxide for 15 min. After washing three times with PBS, slides were incubated in a humidity chamber with 5% donkey serum (Millipore, Billerica, MA, USA) in PBS for 1.5 h at RT. After removal of the blocking solution, slides were incubated with diluted serum samples (1:200 in 5% donkey serum) overnight at 4 °C. The day after, slides were washed three times and incubated with a biotinylated donkey anti-human IgG secondary antibody (Jackson Immuno Research, West Grove, PA, USA) (1:2000, diluted in 5% donkey serum) for 2 h at RT and washed again three times with PBS. An avidin/biotinylated enzyme complex (Vector laboratories, Burlingame, CA, USA) was added for 1 h and subsequently washed three times. Slides were placed in 1:200 diluted Triton X-100 for 30 s and staining was developed with diaminobenzidine (Dako, Glostrup, Denmark) for 7 min. For surface antigen staining, a serum sample positive for α-amino-3-hydroxy-5-methyl-4-isoxazolepropionic acid receptor (AMPAR) antibodies was used as positive control. 

For intracellular antigen screening, rats were perfused with 2% PFA, subsequently decapitated, and the brain dissected. Tissue was fixed in 2% PFA for 4 h at 4 °C and cryoprotected in 20% sucrose for 2–3 days at 4 °C. Isolated cerebellum was embedded in freezing medium and prepared for staining, as explained previously. Each tissue section was incubated for 30 min with blocking solution (10% donkey serum in PBS with 0.1% Triton X-100) and then labelled with diluted sera (1:500 in blocking solution) for 3 h at 37 °C in a humidity chamber. Afterwards, slides were washed twice with PBS and incubated with biotinylated donkey anti-human IgG (1:4000 in PBS) for 30 min at RT. Thereafter, slides were washed twice with PBS and subsequently incubated with an avidin/biotinylated enzyme complex for 30 min. Slides were washed twice and the enzyme reaction was completed with diaminobenzidine for 5 min. For intracellular antigen staining, serum samples positive for either glutamic acid decarboxylase (GAD) or SOX antibodies were used as positive controls.

### 4.4. Immunocytochemistry

Samples that showed increased reactivity to potassium channel proteins in the protein microarray were tested on commercially available indirect immunofluorescence tests for antibodies against VGKC associated proteins LGI1 and CASPR2 (Euroimmun, Lübeck, Germany). The test was used according to the manual. In brief, LGI1 and CASPR2 transfected HEK293 cells fixed on BIOCHIP slides were incubated with 1:10 diluted serum samples for 30 min at RT and subsequently washed with 0.2% PBS-Tween for 5 min. Serum antibodies were labelled with a fluorescein isothiocyanate (FITC) conjugated anti–human IgG antibody for 30 min under protection of light and washed with 0.2% PBS-Tween for another 5 min. Slides were analysed with a fluorescence microscope (Leica DMI 4000B, Wetzlar, Germany) by at least two investigators (Patrick Peschl, Melanie Ramberger, Markus Reindl).

### 4.5. Enzyme-Linked Immunosorbent Assay (ELISA) to Myelin Antigens

Serum IgG antibodies to the recombinant human MOG extracellular Ig domain (rhMOG, amino acids 1–125), produced in *Escherichia coli* bacteria [27], human myelin basic protein (MBP) purified from human brain [28], and synthetic peptides MOG(1–20), MOG(35–55), MBP(13–32), MBP(83–99), MBP(111–129), MBP(146–170), and proteolipid protein [PLP(139–154)] were analysed by ELISA, as described previously [16,29,30].

### 4.6. Statistical Analyses

Microarrays were analysed with the Invitrogen ProtoArray^®^ Prospector Software v5.2.3 (Invitrogen). This software first applies a quantile normalization which is based on the Chebyshev’s inequality principle (CI-P). Therein, an algorithm compares the signal from each protein on the microarray with the signals from the negative control features, to assign a CI-P value. Each signal is then compared to the mean value and the standard deviation of all the signals coming from all proteins spotted on the array resulting in a z-score, indicating the signal strength of each protein. Median values of each z-score duplicate were assessed and a quantile of all signal intensities was evaluated. Only protein signals with z-scores above the 1% top quantile of 2.33 were considered significant hits. Between-group comparisons were performed using matched two-way ANOVA. Statistical analysis and drawing of figures was performed using GraphPad PRISM 7 (GraphPad Software Inc., La Jolla, CA, USA).

## 5. Conclusions

In conclusion, we found antibody reactivity to several candidate proteins in MOG antibody seronegative ADEM patients, but the methods used here comprise several limitations. Bearing in mind the importance of novel biomarkers for therapeutic and prognostic purposes, other methods for antibody screening should be used in future studies. In particular, the preservation of original conformational epitopes should be considered.

## Figures and Tables

**Table 1:** Protein sequences with z-scores ≥ 2.33 in at least two patients within the myelin oligodendrocyte glycoprotein (MOG) antibody negative patient group. Listed proteins were shown to be autoantibody target in at least two samples per group; protein reactivity of only one patient per group was not included in this list. Source: uniprot.org.

Protein Symbol	Protein Name and Known Function	MOG Antibody Negative	MOG Antibody Positive
MLLT6	Myeloid/lymphoid or mixed-lineage leukaemia, translocated to, 6	4	1
RDBP	RNA binding protein, component of negative elongation factor E (NELF-E) of transcription	3	1
SH2D2A	SH2 domain containing 2A, adaptor protein thought to function in T cell signal transduction	2	0
MYOT	Myotilin, stability of thin filaments during muscle contraction	2	0
PALM2	Paralemmin-2, implicated in plasma membrane dynamics in neurons and other cell types	2	0
C11orf63	Chromosome 11 open reading frame 63, protein not characterized	2	0
LOC283663	Predicted: Homo sapiens hypothetical protein LOC283663, protein not characterized	2	0
KCNAB1 v1/v3	Voltage-gated potassium channel subunit beta-1, variants 1 and 3	2	0
KEAP1	Kelch-like ECH-associated protein 1, regulates antioxidant response and ubiquitination	2	1

## Supplementary Materials

Supplementary materials can be found at www.mdpi.com/1422-0067/18/3/679/s1.

## Abbreviations

ADEM	acute disseminated encephalomyelitis
AMPAR	α-amino-3-hydroxy-5-methyl-4-isoxazolepropionic acid receptor
AQP4	aquaporin-4
CASPR2	contactin-associated protein-2
CI	confidence interval
CI-P	Chebyshev’s inequality principle
CNS	central nervous system
CSF	cerebrospinal fluid
ELISA	enzyme-linked immunosorbent assay
FITC	fluorescein isothiocyanate
GAD	glutamic acid decarboxylase
Ig	Immunoglobulin
IHC	Immunohistochemistry
IPMSSG	International Paediatric Multiple Sclerosis Study Group
KCNAB1	voltage-gated potassium channel subunit beta-1
KCNAB2	voltage-gated potassium channel subunit beta member-2
KCTD6	potassium channel tetramerisation domain containing 6
KCTD18	potassium channel tetramerisation domain containing 18
LGI1	leucine-rich glioma-inactivated 1
MBP	myelin basic protein
MOG	myelin oligodendrocyte glycoprotein
MS	multiple sclerosis
NMO	neuromyelitis optica
NMOSD	NMO spectrum disorders
OD	optical density
PFA	paraformaldehyde
PLP	proteolipid protein
rhMOG	recombinant human MOG
v	Variant
VGKC	voltage-gated potassium channel
