# Supplementary material for: Methodological Challenges in Protein Microarray and Immunohistochemistry for the Discovery of Novel Autoantibodies in Paediatric Acute Disseminated Encephalomyelitis
Source: Int J Mol Sci. 2017 Mar 22;18(3):679. doi: 10.3390/ijms18030679 (PMC5372689; doi:10.3390/ijms18030679)
Supplement: Supplementary file 1 [file ijms-18-00679-s001.pdf]

Supplementary Materials: Methodological Challenges in Protein Microarray and Immunohistochemistry for the Discovery of Novel Autoantibodies in Paediatric Acute Disseminated Encephalomyelitis

Patrick Peschl, Melanie Ramberger, Romana Höftberger, Karin Jöhrer, Matthias Baumann, Kevin Rostásy and Markus Reindl

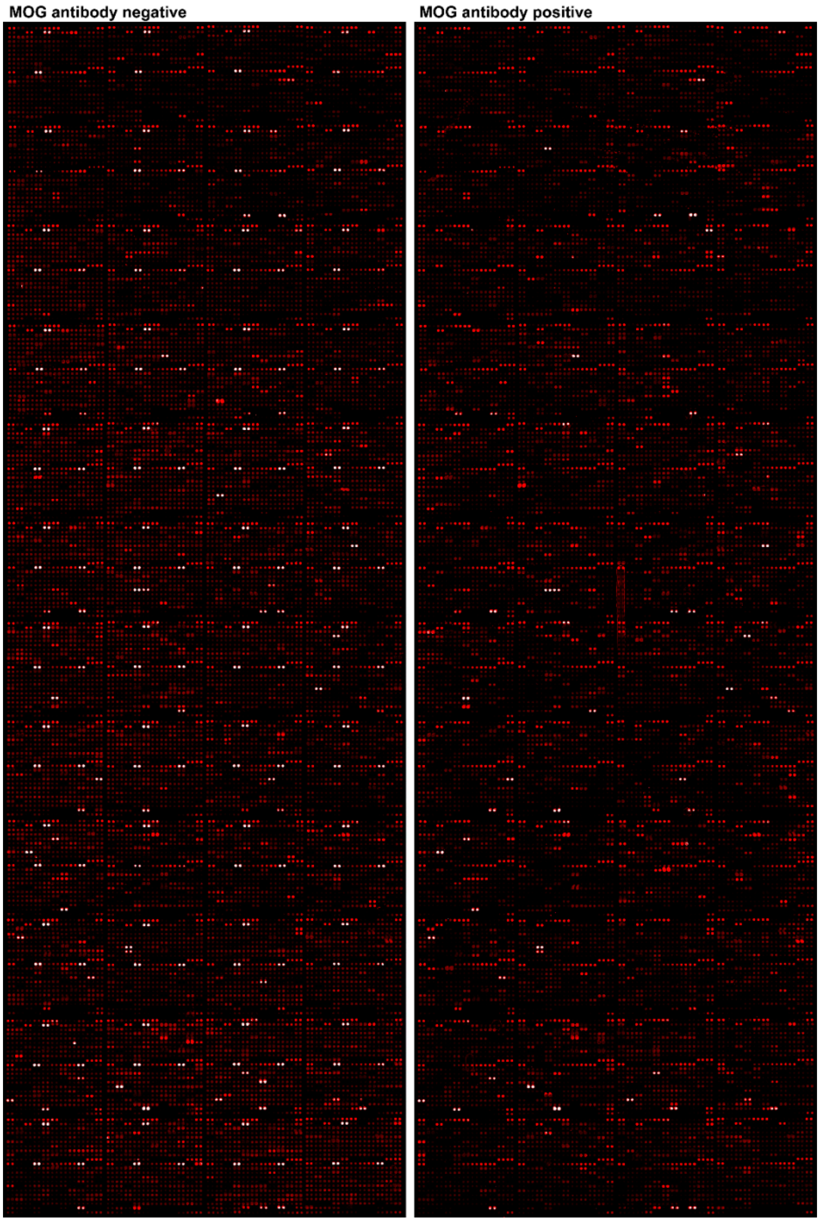

Figure S1. Representative example of protein microarray. Each red dot indicates a single protein on the slide. Left: myelin oligodendrocyte glycoprotein (MOG) antibody negative patient, right: MOG antibody positive patient.

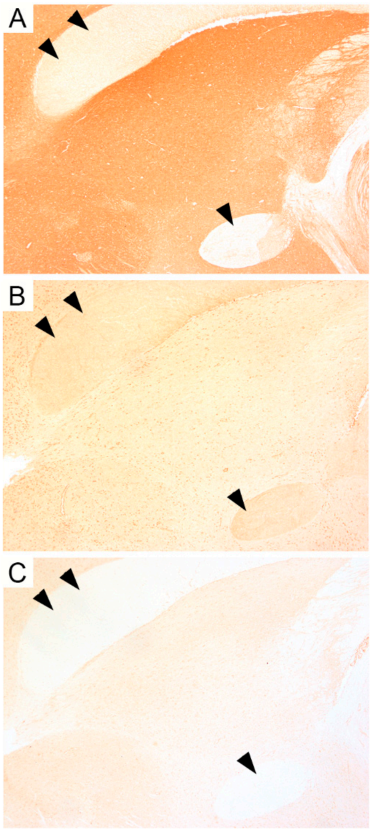

Figure S2. Patient´s serum with α-amino-3-hydroxy-5-methyl-4-isoxazolepropionic acid receptor (AMPAR) antibodies strongly labels the basal ganglia while the white matter (arrows: corpus callosum and commissura anterior) is spared (**A**), in contrast, patient's serum with MOG antibodies clearly labels the white matter (arrows) and the basal ganglia are spared (**B**); serum from a healthy individual is negative (**C**). Magnification: A–C: ×40.

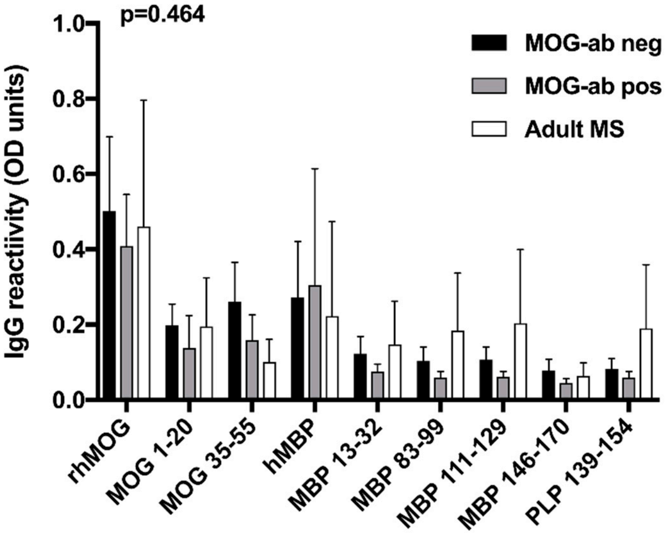

Figure S3. Myelin peptide/protein enzyme-linked immunosorbent assay (ELISA): no significant difference in matched two-way ANOVA (data shown as means with 95% confidence interval) between MOG antibody negative acute disseminated encephalomyelitis (ADEM) patients (11), MOG antibody positive ADEM patients (5) and adult multiple sclerosis (MS) patients (10) [15] for all peptides and recombinant proteins. OD, optical density
